# The SARS Coronavirus S Glycoprotein Receptor Binding Domain: Fine Mapping and Functional Characterization

**DOI:** 10.1186/1743-422X-2-73

**Published:** 2005-08-25

**Authors:** Samitabh Chakraborti, Ponraj Prabakaran, Xiaodong Xiao, Dimiter S Dimitrov

**Affiliations:** 1 Protein Interactions Group, LECB, CCR, NCI-Frederick, NIH, Frederick, MD 21702-1201

## Abstract

The entry of the SARS coronavirus (SCV) into cells is initiated by binding of its spike envelope glycoprotein (S) to a receptor, ACE2. We and others identified the receptor-binding domain (RBD) by using S fragments of various lengths but all including the amino acid residue 318 and two other potential glycosylation sites. To further characterize the role of glycosylation and identify residues important for its function as an interacting partner of ACE2, we have cloned, expressed and characterized various soluble fragments of S containing RBD, and mutated all potential glycosylation sites and 32 other residues. The shortest of these fragments still able to bind the receptor ACE2 did not include residue 318 (which is a potential glycosylation site), but started at residue 319, and has only two potential glycosylation sites (residues 330 and 357). Mutation of each of these sites to either alanine or glutamine, as well as mutation of residue 318 to alanine in longer fragments resulted in the same decrease of molecular weight (by approximately 3 kDa) suggesting that all glycosylation sites are functional. Simultaneous mutation of all glycosylation sites resulted in lack of expression suggesting that at least one glycosylation site (any of the three) is required for expression. Glycosylation did not affect binding to ACE2. Alanine scanning mutagenesis of the fragment S319–518 resulted in the identification of ten residues (K390, R426, D429, T431, I455, N473, F483, Q492, Y494, R495) that significantly reduced binding to ACE2, and one residue (D393) that appears to increase binding. Mutation of residue T431 reduced binding by about 2-fold, and mutation of the other eight residues – by more than 10-fold. Analysis of these data and the mapping of these mutations on the recently determined crystal structure of a fragment containing the RBD complexed to ACE2 (Li, F, Li, W, Farzan, M, and Harrison, S. C., submitted) suggested the existence of two hot spots on the S RBD surface, R426 and N473, which are likely to contribute significant portion of the binding energy. The finding that most of the mutations (23 out of 34 including glycosylation sites) do not affect the RBD binding function indicates possible mechanisms for evasion of immune responses.

## Background

Viral envelope glycoproteins initiate entry of viruses into cells by binding to cell surface receptors followed by conformational changes leading to membrane fusion and delivery of the genome to the cytoplasm [1]. The spike (S) glycoproteins of coronaviruses are no exception and mediate binding to host cells followed by membrane fusion; they are major targets for neutralizing antibodies and form the characteristic corona of large, distinctive spikes in the viral envelopes [2,3]. Such 20 nm complex surface projections also surround the periphery of the SCV particles [4]. The level of overall sequence similarity between the predicted amino acid sequence of the SCV S glycoprotein and the S glycoproteins of other coronaviruses is low (20–27% pairwise amino acid identity) except for some conserved sequences in the S2 subunit [5]. The low level of sequence similarity precludes definite conclusions about functional and structural similarity.

The full-length SCV S glycoprotein and various soluble fragments have been recently cloned, expressed and characterized [6-11]. The S glycoprotein runs at about 170–200 kDa in SDS gels suggesting posttranslational modifications as predicted by previous computer analysis and observed for other coronaviruses [6,11]. S and its soluble ectodomain, Se, were not cleaved to any significant degree [6]. Because the S protein of coronaviruses is a class I fusion protein [12], this observation classifies the SCV S protein as an exception to the rule that class I fusion proteins are cleaved exposing an N-terminal fusogenic sequence (fusion peptide) although cleavage of S could enhance fusion [9].

Because S is not cleaved, it is difficult to define the exact location of the boundary between S1 and S2; presumably it is somewhere between residues around 672 and 758 [6,7]. Fragments containing the N-terminal amino acid residues 17 to 537 and 272 to 537 but not 17 to 276 bound specifically to Vero E6 cells and purified soluble receptor (ACE2) molecules [6]. Together with data for inhibition of binding by antibodies, developed against peptides from S, these findings suggested that the receptor-binding domain (RBD) is located between amino acid residues 303 and 537 [6]. Two other groups obtained similar results and found that independently folded fragments containing residues 318 to 510 [8] and 270 to 510 [10] can bind receptor molecules. Currently, these fragments are being further characterized to better understand the interactions of the virus with its receptor as well as their potential as inhibitors of the virus entry by blocking these interactions. Here, we present evidence that glycosylation of these and other fragments containing the S RBD does not affect to any measurable degree their binding to the receptor (ACE2), and analyze the S RBD-ACE2 interaction.

## Results

### A short RBD fragment containing only two potential glycosylation sites folds independently and binds ACE2

**Figure 1:**
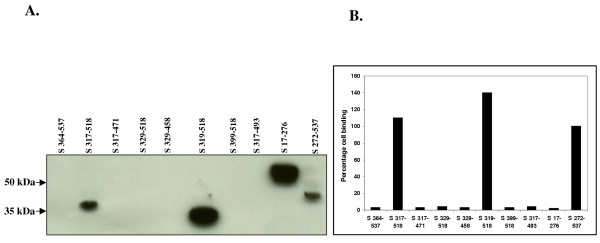
**Expression and binding of soluble S fragments containing the RBD. **A) Soluble S proteins concentrated using Ni-NTA agarose beads from the supernatants of 293 cells transfected with various constructs were run, blotted onto a nitrocellulose membrane and detected with anti-c-myc epitope antibody. B) Cell binding assay data using supernatants described above, shown as a percentage of the reading of S272–537 that has been used in this experiment as a positive control.

We and others have previously identified the RBD by using fragments containing three potential glycosylation sites – at residues 318, 330 and 357 [6,8,10]. To find the minimal number of potential glycosylation sites and shortest length required for expression and folding of S RBD fragments, we cloned in pSecTag 2B fragments with various number of potential glycosylation sites and length including S317–518, S319–518, S329–518, S364–537, S399–518, S317–493, and S329–458, where the numbers after S denote the amino acid residues confining the fragment. Note that these fragments were not constructed as fusion proteins with Fc as in a previous report [8]. This is why we also designed and tested several fragments with deleted portions of the RBD that have already been shown to be important for binding to ACE2 including regions between residues 327 and 490 [8]. The S317–518 and S319–518 fragments were secreted in the culture supernatant (Fig 1A), and bound to ACE2-expressing cells (Fig 1B) and purified ACE2 (Table 1 and data not shown). The difference in the molecular weights of the two fragments (about 3 kD) is much larger than the calculated weight due to the two additional amino acids contained in S317–518, and is likely due to glycosylation. Both fragments bound to ACE2 at comparable levels (Fig. 1B). The other fragments were not secreted (Fig. 1A) but could be detected by Western in cell lysates (data not shown). These results suggest that a short fragment (S319–518), which is not a fusion protein, with only two glycosylation sites can be independently folded and secreted in a soluble form, and can bind ACE2.

**Table 1:** S RBD mutants, expression levels and binding to ACE2.

Mutant	Mutation	Expression	Binding	ASA
1	E327	98	83	123
2	K333	86	90	176
3*	K344	95	102	159
**4**	**K390**	**104**	**1**	**44**
5	D392	110	95	69
6	D393	30	100	10
7	K411	90	103	33
8	D414	120	130	113
9	D415	90	102	97
10*	**R426**	**73**	**7**	**95**
11	N427	100	111	121
**12**	**D429**	**103**	**0**	**9**
**13**	**T431**	**131**	**64**	**59**
14	K439	85	87	65
15	R441	10	15	3
16*	Y442	105	110	68
17*	R444	80	86	52
18	H445	124	103	113
19	K447	87	85	138
20	R449	96	101	178
21	F451	69	71	64
**22**	**D454**	**50**	**4**	**25**
**23**	**I455**	**77**	**6**	**89**
24	D463	87	81	70
25*	L472	95	99	172
**26**	**N473**	**100**	**0**	**70**
27	W476	80	76	126
**28**	**F483**	**91**	**3**	**2**
**29**	**Q492**	**95**	**3**	**5**
**30**	**Y494**	**50**	**7**	**21**
**31**	**R495**	**97**	**19**	**7**
32	E502	110	84	175
**33**	**S17–276**	**90**	**0**	
34	S319–518	100	100	

The mutants that significantly decrease binding to ACE2 are shown in bold. The * denotes mutant residues that are naturally occurring in various SCV strains (see Fig. 6A). The binding and expression values for the individual mutants are expressed as a percentage of the value for the S319–518 (wt) that is assumed 100%. The values of accessible surface area (ASA, Å^2^) for mutant residues were calculated from the crystal structure of the S RBD-ACE2 complex (coordinates provided by S. Harrison) by using the Lee and Richards' algorithm [23] with a probe radius of 1.4 Å.

### The potential glycosylation sites in RBD fragments are functional and glycosylation does not affect binding to ACE2

**Figure 2:**
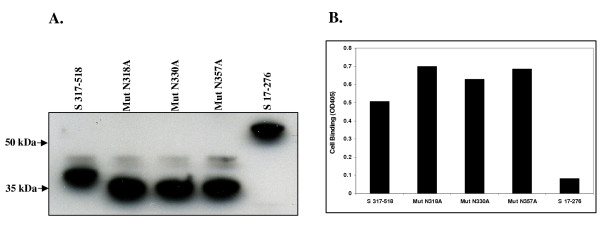
**Glycosylation of S fragment containing the RBD. **A) Expression of the three mutants on S317–518 where the potential sites of glycosylation at N318, N330 and N357 were individually converted to alanine. All the mutants appear to have similar molecular weights when compared to the wild type protein S317–518. B) Cell binding data of the same mutants.

**Figure 3:**
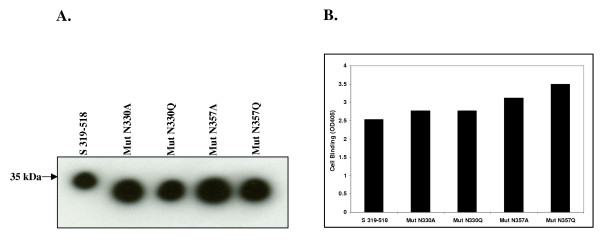
**Effects of glycosylation on expression and binding of RBD-containing fragments. **A) Expression of the four mutants on S319–518 where the two sites of glycosylation at N330 and N357 have been individually converted to either alanine or glutamine. The various mutants have similar molecular weights, a little less than the wild type indicating that the level of glycosylation at each residue might be similar. B) Cell binding data for the same mutants.

To find whether the potential glycosylation sites in the RBD fragments are functional we constructed mutants, where the three residues N318, N330 and N357 in S317–319 were mutated individually from asparagine to alanine. As is shown in Fig. 2A all three mutants were expressed and ran on SDS-PAGE at molecular weights of about 3 kD smaller than the unmodified fragment. They all bound to ACE2 (Fig. 2B). Similar results were obtained with the shorter fragment (S319–518) where asparagines were also mutated to glutamines, which better mimic asparagines (Fig. 3). These results suggest that all glycosylation sites in the RBD are functional, and that the lack of glycosylation in any of the glycosylation sites does not interfere with binding to ACE2.

### Only one glycosylation site is required for secretion of functional RBD fragments

**Figure 4:**
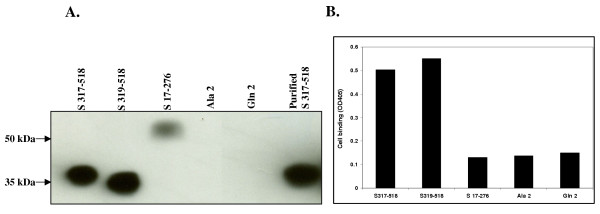
**Glycosylation of at least one residue in RBD-containing fragments is required for expression. **A) Expression pattern of two mutants on S319–518 in which both the glycosylation sites at N330 and N357 have been mutated either to alanine or to glutamine. No expression is seen when both the sites have been mutated indicating that glycosylation of at least one of the sites is important. In the last lane, purified S317–518 protein has been loaded as a control. B) Cell binding results of the same mutants.

To find the minimal number of functional glycosylation sites required for secretion of the RBD we generated double mutants of S319–518 where the asparagines N330 and N357 were mutated to either alanines (Ala 2) or glutamines (Gln 2). These mutants were not detected in the culture supernatants (Fig. 4A) and the culture supernatants did not exhibit any binding activity to ACE2 (Fig. 4B). These results suggest that at least one glycosylation site is required for secretion of functional RBD fragments.

### Identification of 11 RBD amino acid residue mutations that affect its binding to ACE2, and 20 – that do not

To identify RBD amino acid residues that might affect binding to ACE2, we converted 32 residues in S319–518 to alanine, expressed the mutants and tested their binding to ACE2. Eleven mutants, K390, R426, D429, T431, D454, I455, N473, F483, Q492, Y494, and R495 exhibited decreased binding to ACE2 at comparable levels of expression (Table 1). Note that RBD fragment mutated at D454 or Y494 was expressed at somewhat lower levels but binding was much more significantly reduced. In addition, one of these mutations, D454, was previously shown to affect the RBD-ACE2 interaction [8]. The T431 mutation reduced binding but to lesser extent than the other mutations that decreased very significantly (more than 10-fold) the RBD-ACE2 interaction. The protein mutated at R441 expressed poorly and we were not able to assess its role in the RBD binding, although because of the similar levels of decrease in binding and expression, it is likely that this mutation does not affect binding. Interestingly, it appears that the D393 mutation enhanced binding – the mutated fragment expressed at low concentration but its binding equaled the binding of the non-mutated protein. The mutated residues that affect RBD binding include positively and negatively charged, polar and hydrophobic residues, indicating a role of electrostatic and hydrophobic interactions in the RBD-ACE2 interactions. These results also demonstrate that the mutations for the selected panel of residues that do affect binding are significantly (about 2-fold) more than those that do not, suggesting possible mechanisms of immune evasion.

### Analysis of the S RBD sequence and the role of critical residues in S RBD

**Figure 5:**
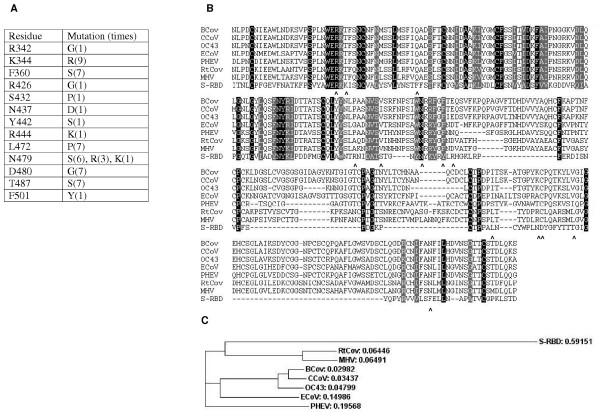
**Multiple sequence alignment of S fragment (RBD) with SARS CoV-related and other coronaviruses/spike glycoproteins. **A) The table shows 13 amino acid residues in the region of S RBD (319–518) which have sequence variations as identified from the multiple sequence alignment of S RBD with 19 SARS CoV-related sequences (97–99% identities with S RBD) using BLAST. B) Multiple sequence alignment of S RBD and 7 other related proteins from different organisms which share 20–35% identities: bovine coronavirus (BCoV, 327–622), canine respiratory coronavirus (CCoV, 327–622), human coronavirus (OC43, 331–612), equine coronavirus (ECoV, 327–622), porcine hemagglutinating encephalomyelitis virus (PHEV, 327–608), rat sialodacryoadenitis coronavirus (RtCoV, 325–610) and murine hepatitis virus (MHV, 325–611). Dark and gray colors indicate the identity and similarity of residues aligned. Arrowheads on the S RBD sequence show the 13 sites, which are found to have sequence variations. C) The phylogram tree is shown with distances along the protein names and note that S RBD has the highest distance. Multiple sequence alignment and phylogram were constructed using ClustalW program.

In order to further characterize the RBD and its interaction with ACE2 we analyzed the sequence and secondary structure, and how they relate to the mutations that affect binding to the receptor. A sequence-based secondary structure analysis of the S RBD predicted mostly β-sheets (data not shown), connected by loops or turns, where most of the residues affecting the RBD-ACE2 interactions are located. To find out additional residues that are not likely to affect binding significantly we aligned multiple RBD sequences of various non-redundant SCV strains. Figure 5A shows the identified 13 amino acid residues, which can be mutated without affecting the function of the virus to cause infection. Interestingly, one of these residues, R426, which decreases binding to ACE2 about 10-fold if mutated to A, is mutated to G in one of the strains. Four of the other 12 mutations (indicated with * in Table 1) do not affect binding to ACE2 when converted to A. To examine the extent of similarities of the SCV RBD sequence with related sequences of other coronaviruses from different organisms, which share only about 20–35% sequence identities, we performed multiple alignments using BLAST. Strikingly, six cysteine residues are conserved (Fig. 5B) indicating the possibility for up to three possible disulphide bridges within the S RBD that can help to keep the structural integrity of this domain. Most of the residues we found important for binding are highly variable except T431, Q492 and R495, which are highly conserved (Fig. 5B). The multiple sequence alignment score was then used to build a phylogram by using the ClustalW software. The results suggested that the SCV S RBD is much more distant than the respective regions of the other tested coronaviruses (Fig. 5C).

**Figure 6:**
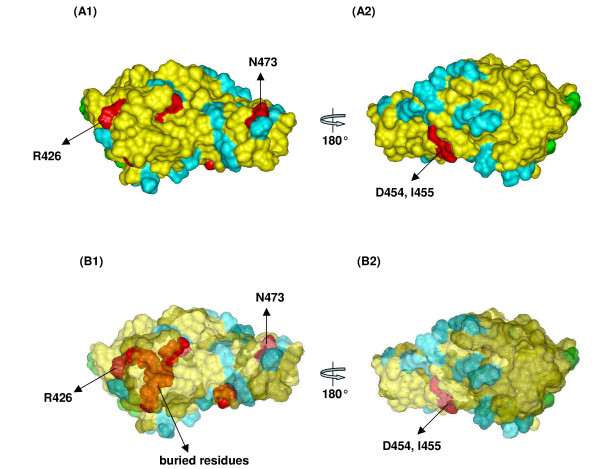
**Mapping of the S RBD mutants on the structure. **The molecular surface diagrams of S RBD are shown as the top views in the solid and translucent models. The S RBD surface is in yellow, mutations that significantly affect the binding to ACE2 are in red and those do not affect the binding in cyan. (A) Shown are the solid surface diagrams using the structure of S RBD (left panel) and related by 180° rotations (right panel). The residues that decrease the receptor binding as observed in the experiment and exposed in the structure are labeled (R426, N473). (B) The same surface diagrams as in A but with transparency which are related by 180° rotations. The buried residues, which reduce the receptor binding as observed in the experiment, are seen as blurred red.

Recently, the crystal structure of S RBD-ACE2 complex was solved and the coordinates became available after the completion of this study, kindly provided by Stephen Harrison (Li, F, Li, W, Farzan, M, and Harrison, S. C., submitted). We have mapped the S RBD mutations on the surface of the crystal structure by using InsightII software. The Connolly molecular surface of the S RBD as viewed from the receptor ACE2 is shown in Fig. 6A. The S RBD is in yellow color in which the mutants that significantly affect the binding to ACE2 are shown in red and those that do not affect the binding are in cyan. The two glycosylation sites at 330 and 357 positions are colored in green. In the right panel the structure is rotated by 180° to show the opposite side of the RBD surface.

In the structure of the S RBD-ACE2 complex two of the mutants with very significantly reduced binding to ACE2, R426A and N473A, make contacts with ACE2 residues and are completely exposed (Table 1). They are separated by residues whose mutations do not affect the S RBD binding to ACE2. Interestingly, six of the mutations we identified to reduce binding are buried but at close proximity to R426 as shown by the translucent surface highlighting in Fig. 6B indicating sensitivity of this area to mutations and likely involvement of other residues. Residues D454 and I455, whose mutation reduced binding to ACE2, do not make contacts with ACE2 and are located on the side opposing the side facing the receptor (right panel of Fig. 6); it is likely that the mutations decrease binding by inducing conformational changes. Other mutations including mutations of the two glycosylations sites on that side do not affect binding to ACE2 (right panels of Fig. 6). These results suggest the existence of two hot spots on the S RBD surface, R426 and N473, which are likely to contribute significant portion of the binding energy.

## Discussion

The major results of this work are the demonstration of the functionality of the potential glycosylation sites of the S RBD and the requirement of at least one of them for its proper expression as well as the identification of two hot spots on the S RBD surface, R426 and N473, which are likely to contribute significant portion of the binding energy to ACE2. ACE2 was previously identified as a receptor for the SCV [7] and this finding was confirmed [6,13]. ACE2 binds with high (nM) affinity to S and is expected to induce conformational changes required for membrane fusion [6-8,14]. Its crystal structure was recently reported [15] and is in general agreement with two homology models previously developed [16,17]. It was proposed that the S binding domain on ACE2 involves residues on the ridges surrounding the enzymatic site [17]. Recently, several ACE2 regions and amino acid residues were identified as important for its binding to the S RBD [18].

Currently, the three-dimensional (3D) structure of the S RBD in free unbound form is unknown. We performed sequence analysis and developed a 3D model of a fragment containing the S RBD (the model will be described elsewhere). According to this model the S RBD like RBDs from other viruses contains predominantly β-sheets. Most of the residues affecting the ACE2 interactions are exposed on the surface of the beta sheets and inter-connecting loops. These predicted observations are consistent with the recently solved crystal structure of S RBD complexed with ACE2 (Li, F, Li, W, Farzan, M, and Harrison, S. C., submitted). The nature of the residues, which include charged, hydrophobic and polar residues indicated that all these types of interactions could be involved either directly or indirectly in the S RBD binding to ACE2. Notable are the complementarities in the charges of several residues in S, e.g. R426 and N473 with those of ACE2, e.g. E329 and Q24, respectively. One can reason that these residues might contribute significantly for the on rate constant and proper orientation of the two molecules in the complex, as well as to the low dissociation rate constant. We identified two hot spots, residues R426 and N473, which are likely to contribute to the bulk of the free energy of interaction. Further studies are required for the elucidation of the energy profile of the S RBD-ACE2 interaction.

We found that not only glycosylation of the three sites in the previously described RBD-containing fragments is dispensable for expression (except one that can be any) but it also does not affect binding to ACE2. Indeed all glycosylation sites are localized at the N-terminal portion of the RBD and are relatively close to each other not only in the sequence (residues 318, 330 and 357) but also in the 3D space (Fig. 6). We constructed a fragment (319–518), which contains only two glycosylation sites and still binds with an affinity undistinguishable from the fragments containing three glycosylation sites. Further mutations of all combinations of these sites revealed that only one of them is required for expression but none of them for binding. Therefore the S RBD contacts ACE2 by an area lacking carbohydrates, which is in agreement with the recently solved crystal structure of the S RBD (Li, F, Li, W, Farzan, M, and Harrison, S. C., submitted).

The entry of the SCV into cells can be inhibited by antibodies that bind the S glycoprotein and prevent its binding to ACE2. Such a monoclonal antibody that potently inhibits membrane fusion at nM concentrations was recently identified by screening phage display libraries [19]. This antibody competed with ACE2 for binding to the S glycoprotein suggesting that its mechanism of neutralization involves inhibition of the virus-receptor interaction. We have also identified several antibodies specific for the S RBD ([20] and Zhu and Dimitrov, in preparation). The mutants developed in this study could be useful for mapping the epitopes of the antibodies against the S RBD, most of which are likely to neutralize the virus by preventing binding to the receptor ACE2.

Most of the mutations (20) described in this study did not affect binding of the S RBD to ACE2. This finding suggests that the virus could easily mutate and escape antibodies that do not exhibit the same energy profile of binding to S as ACE2. However, further studies are required in the context of the whole oligomeric S protein to make more definite conclusions about possible mechanisms of immune evasion.

The results reported in this study could have implications for understanding the mechanisms of SCV entry, and for development of entry inhibitors, vaccine immunogens, and research tools. Future studies particularly the solution of the crystal structure of the S protein in free unbound form, and in complex with ACE2, as well as measurements of the energy profiles of binding to ACE2 and antibodies, could elucidate detailed mechanisms of the S RBD function that may help in the further development of clinically useful inhibitors and vaccines.

## Methods

### Plasmids and antibodies

Plasmid encoding the soluble form of ACE2, pCDNA3-ACE2-ecto, was kindly provided by M. Farzan from Harvard Medical School, Boston, Massachusetts. VTF7.3 is a kind gift from C. Broder, USUHS, Bethesda, MD. Expression vectors pSecTag2 series were purchased from Invitrogen (Carlsbad, California). The monoclonal anti-c-Myc epitope antibodies (unconjugated and conjugated to HRP) were obtained from Invitrogen (Carlsbad, CA).

### Cloning of S fragments

Using the previously described S756 [6] plasmid as template, fragments S364–537 (5'-GATCGGATCCTCAACCTTT AAGTGC-3' and 5'-GATCGAATTCC AGTAC CAGTGAG-3'), S317–518 (5'-GATCGGATCCCCTAATATTACAAAC-3' and 5'-G ATCGAATTCGGTCAGTGG-3'), S317–471 (5'-GATCGGATCC CCTAATATTAC AAAC-3' and 5'-GATCGAATTCGAGCAGGTGGG-3'), S329–518 (5'-GATCGGA TCCTTCCC TTCTGTC-3' and 5'-GATCGAATTCG GTCAGTGG-3'), S329–458 (5'-GATC GGATCCTTCCCTTCTGTC-3' and 5'-GATCGAATTCGCACATTAGA TATGTC-3'), S319–518 (5'-GATCGGATCCA TTACAAACTTGTGTCC-3' and 5'-GATCGAATTCG GTCAGTGG-3'), S399–518 (5'-GATCGGATCCCCAGG ACAA ACTGG-3' and 5'-GA TCGAAT TCGGTCAGTGG-3'), and S317–493 (5'-GATCG GATCCCCTAATATTACA AAC-3' and 5'-GATCGAATTCAAGG TTGGTAGCC-3') were PCR amplified using the primers mentioned within the parentheses. The PCR amplified fragments were then directionally cloned into expression vector pSecTag 2B using the restriction enzymes Bam HI and Eco RI. The various mutations on S317–518 and S319–518 were generated using the QuickChange^® ^XL Site Directed Mutagenesis kit (Stratagene, La Jolla, CA) following the manufacturer's protocol.

### Protein expression

Various plasmids were transfected into 293 cells using the Polyfect transfection kit from Qiagen (Valencia, CA) following the manufacturer's protocol. Four hours after transfection, cells were infected with VTF7.3 recombinant vaccinia virus encoding the gene for the T7 polymerase. The soluble S fragments were obtained from the cell culture medium.

### Western blotting

Loading buffer and DTT (final concentration 50 mM) were added to either S proteins concentrated from the culture supernatant using Ni-NTA agarose beads or directly to the supernatant, boiled and run on an SDS-PAGE. The monoclonal anti-c-Myc epitope antibody (Invitrogen, Carlsbad, CA) was diluted in TBST buffer and incubated with the membrane for 2 hours, washed and then incubated with the secondary antibody conjugated with HRP for 1 hour, washed four times, each time for 15 min, and then developed using the ECL reagent (Pierce, Rockford, IL).

### Cell binding assay

Medium containing soluble S fragments was collected and cleared by centrifugation. Vero E6 cells (5 × 10^6^) were incubated with 0.5 ml of cleared medium containing soluble S fragments and 2 μg of anti-c-Myc epitope antibody conjugated with HRP at 4°C for two hours. Cells were then washed three times with ice cold PBS and collected by centrifugation. The cell pellets were incubated with ABTS substrate from Roche (Indianapolis, IN) at RT for 10 min., the substrate was cleared by centrifugation, and OD405 was measured.

### ELISA

For the detection of the S protein fragments, a sandwich ELISA was used in which the plate was coated with anti-His tag antibody. The S protein containing culture supernatants were added and detected with an anti-c-Myc epitope antibody. In the second ELISA, the S protein was bound to the C9-tagged ecto-domain of receptor ACE 2 that was captured on a plate coated with anti-C9 antibody (ID4). As in the previous ELISA, the S protein was detected with anti-c-myc epitope antibody. The second ELISA was used to score the binding of the various S protein fragments to the receptor ACE 2. In all experiments, the incubations with the c-myc epitope antibody were for 2 h at RT.

### Sequence analysis of S RBD

Sequence similarity searches were performed using NCBI BLAST program [21] by selecting, separately, all non-redundant sequences (nr) and sequences derived from the 3-dimensional structure records from the Protein Data Bank (PDB). The BLAST analysis against nr database showed 19 SARS CoV-related sequences from different clones with identities of 97–99% from the top of the list as well as 7 different coronaviruses from other organisms which share only 20–35% sequence identities at the bottom. These sequences were collected and aligned with the sequence of SARS RBD fragment using ClustalW program [22] with default parameters. The multiple alignment sequence table was prepared by choosing the aligned sequences with optimal gaps and then a phylogram tree was constructed based on that alignment scores for the 7 different coronaviruses along with S RBD. Further, the BLAST against PDB database retrieved 5 hits and 4 of them have longer stretch of amino acids (PDB codes: 1KS5, 1K0H, 1NKG and 1QR0), which have detectable sequence similarities with different regions of SARS RBD.

## Competing interests

The author(s) declare that they have no competing interests.
